# Drive shaft sheath may work as guide extension catheter in rotational atherectomy

**DOI:** 10.1007/s12928-026-01272-4

**Published:** 2026-05-29

**Authors:** Kenichi Sakakura, Hiroyuki Jinnouchi, Hideo Fujita

**Affiliations:** Division of Cardiovascular Medicine, Saitama Medical Center, Jichi Medical University, 1-847 Amanuma, Omiya, Saitama City, 330-8503 Japan

Rotational atherectomy (RA) has been used for severely calcified coronary lesions for ≥ 20 years. In RA, we usually call the start position of the burr within coronary arteries as “platform” [1]. Operators advance the burr from the platform to the target lesion in each session and bring back the burr to the platform. The drive shaft sheath (DSS), which is a transparent and radiolucent tube, is a key component of ROTAPRO (Boston Scientific, Marlborough, USA). Pressurized normal saline or cocktail reaches coronary arteries via the DSS to prevent heat injury or slow flow.

Recently, guide extension catheters (GEC) have been developed for complex coronary lesions [2]. When we use GEC for stent delivery, it is better to advance GEC as distal as possible, partly because GEC prevent the wobbling of the stent delivery system. Since the DSS is somewhat similar to GEC, we hypothesized that the DSS prevents the wobbling of the drive shaft, which would be important for stable burr motion. We performed a simplified bench test using ROTAPRO, RotaWire Drive Floppy, Mach 1 7Fr JL 3.5 (Boston Scientific, Marlborough, USA), and 2-D model. A pseudo-lesion composed by styrene was used in 2-D model. We recorded the burr motion in two different knob platform positions. In the proximal knob platform position [the knob is the most proximal (right) end], the distance between the tail of the burr and the tip of DSS is approximately 14 mm (Fig. 1A). We activate the burr with 160,000 rpm and advance the burr with approximately 2 cm working range (Fig. 1B and C, Supplemental Movie 1). The drive shaft seemed to be stable without wobbling (Fig. 1C, Supplemental Movie 1). In the distal knob platform position [the knob is approximately 2 cm before the distal (left) end], the distance between the tail of the burr and the tip of DSS is approximately 68 mm (Fig. 1D). We activate the burr with 160,000 rpm and advance the burr with approximately 2 cm working range (Fig. 1E and F, Supplemental Movie 1). The drive shaft showed wobbling motion (Fig. 1F, Supplemental Movie 1).

**Fig. 1:**
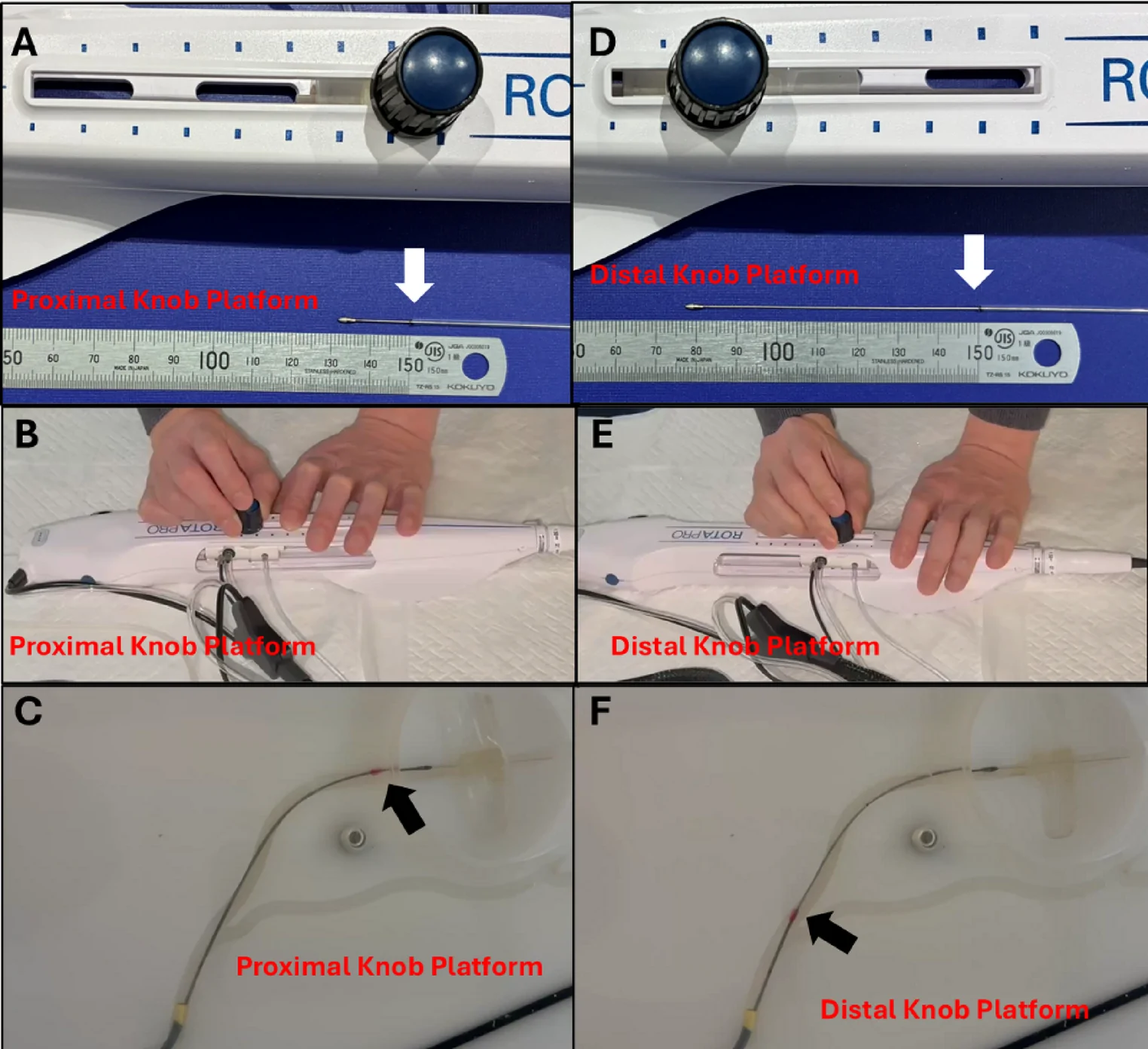
Difference between proximal and distal knob platform. **A**. The knob was positioned at the most proximal (right) end. Arrow indicates the tip of DSS (inked black). **B**. The burr was activated at 160,000 rpm within approximately 2 cm working range. **C**. The drive shaft seemed to be stable without wobbling. Arrow indicates the tip of DSS (inked red). **D**. The knob was positioned at 2 cm before the distal (left) end. Arrow indicates the tip of DSS (inked black). **E**. The burr was activated at 160,000 rpm within approximately 2 cm working range. **F**. The drive shaft showed wobbling motion. Arrow indicates the tip of DSS (inked red)

Our bench test showed that the DSS partly may work as GEC to prevent the wobbling of the drive shaft. Because the tip of the DSS is completely invisible in coronary arteries, operators cannot confirm the accurate position of the tip of the DSS. Operators can only imagine the position of the tip of the DSS by the knob position. If the knob is very distal (very left), the tip of the DSS is very far from the burr. In such situation, the burr control may be difficult due to the wobbling of the drive shaft. When RA is applied to diffuse long lesions, we usually move the platform forward between sessions. However, in most cases, we tend to advance the platform by only advancing the knob position but do not advance the tip of DSS, partly because the tip of DSS is invisible in fluoroscopy. If we advance the platform without considering the tip of DSS, the distance between the burr and the tip of DSS may be very far, which may weaken the penetration force. In such situations, we may care about the knob position (the tip of DSS) as well as the burr position for safer RA procedures, especially when operators have difficulty to advance the burr beyond the distal target. Furthermore, if the distance between the burr and the tip of DSS is very far, the wobbling of the drive shaft itself may cause adverse events such as coronary spasm. The pressurized cocktail including vasodilators may not reach to the debulking lesion, which may result in excessive heat production, coronary spasm, or slow flow. Finally, other factors such as rotational speed may influence shaft vibration, torque transmission, and burr stability. Dedicated bench tests are warranted for further refinement in RA procedures.

## Supplementary Information

Below is the link to the electronic supplementary material.

Supplementary Material 1. Difference between proximal and distal knob platform. **A**. The knob was positioned at the most proximal (right) end. Arrow indicates the tip of DSS (inked black). **B**. The burr was activated at 160,000 rpm within approximately 2 cm working range. **C**. The drive shaft seemed to be stable without wobbling. Arrow indicates the tip of DSS (inked red). **D**. The knob was positioned at 2 cm before the distal (left) end. Arrow indicates the tip of DSS (inked black). **E**. The burr was activated at 160,000 rpm within approximately 2 cm working range. **F**. The drive shaft showed wobbling motion. Arrow indicates the tip of DSS (inked red)
